# Intramedullary injection of polymerized laminin in acute traumatic spinal cord injury: a first-in-human pilot study

**DOI:** 10.1038/s41393-026-01238-6

**Published:** 2026-08-04

**Authors:** Marco Aurelio B. Lima, Karla Menezes, Denise R. Xerez, Bruno A. Côrtes, João R. L. Menezes, Gustavo S. Holanda, Adriana D. Silva, Eliel S. Leite, Olavo B. Franco, Marco Aurélio A. M. de Faria, Arthur de Freitas Forte, Livia V. Abreu, Renata C. L. Lichtenberger, Cíntia F. Santana, Maurilio Rosa, Aurélio V. Graça-Souza, Álvaro U. C. Jorge, Ana Cristina Franzoi, Arthur S. Ferreira, Rose M. Frajtag, Tatiana Coelho‐Sampaio

**Affiliations:** 1 Institute of Biomedical Sciences, Federal University of Rio de Janeiro, Rio de Janeiro, Brazil; 2 Department of Neurosurgery, Public State Hospital Azevedo Lima, Niteroi, Brazil; 3 Department of Internal Medicine, Federal University of Rio de Janeiro, Rio de Janeiro, Brazil; 4 Department of Neurosurgery, Rio de Janeiro Municipal Hospital Souza Aguiar, Rio de Janeiro, Brazil; 5 National Cancer Institute, Rio de Janeiro, Brazil; 6 Institute of Medical Biochemistry, Federal University of Rio de Janeiro, Rio de Janeiro, Brazil; 7 Augusto Motta University Center, Rio de Janeiro, Brazil

**Keywords:** Drug development, Translational research, Spinal cord injury

## Abstract

**Study design:**

Investigator-initiated, single-arm, first-in-human pilot study.

**Objectives:**

To evaluate the safety and feasibility of intramedullary administration of polymerized laminin in individuals with acute complete spinal cord injury (SCI).

**Setting:**

Public and private hospitals in Brazil.

**Methods:**

Eight patients with acute traumatic SCI classified as AIS A (C4–T12) were enrolled within days after injury (mean 2.3 days). Polymerized laminin was administered as a single intramedullary dose (1 µg/kg; total 55–80 µg). Patients were followed for 12 months using standardized neurological, laboratory, and neurophysiological assessments.

**Results:**

Intramedullary administration was uneventful in all cases. No neurological deterioration or serious adverse events attributable to the intervention were observed. Mild laboratory abnormalities were transient and not associated with hepatic or renal toxicity. Three participants died during follow-up. Deaths were reviewed by the Brazilian National Research Ethics Commission (CONEP) and an external clinical consultant, and were not considered related to the intervention. Neurological improvement of at least two AIS grades was observed in six participants, including one who died after progressing to AIS C. Improvements in motor and/or somatosensory evoked potentials were observed in three patients.

**Conclusions:**

Intramedullary administration of polymerized laminin was uneventful and appeared to be safe in this small and heterogeneous cohort. Although the study design does not allow conclusions regarding efficacy, it provides proof-of-concept for this therapeutic approach and justifies further evaluation in controlled clinical studies. The study was initiated in December 2016, prior to current requirements for prospective trial registration.

## Introduction

Spinal cord injury (SCI) remains an unmet medical need, with limited therapeutic options capable of promoting meaningful neurological recovery. The pathophysiology of central nervous system injury is complex and multifactorial, involving overlapping processes such as inflammation, axonal disruption, demyelination, and glial scar formation. This complexity suggests that effective therapeutic strategies may require interventions acting on multiple biological pathways rather than targeting a single mechanism [1, 2]. Consistent with this, spontaneous recovery after complete SCI is generally limited, particularly with respect to restoration of voluntary motor function below the level of injury [3–5].

Laminin, a key component of the extracellular matrix, is of particular interest in this context due to its broad regulatory effects on the nervous system [6–10]. During peripheral nerve regeneration, axons grow within laminin-rich conduits formed by Schwann cells [11]. In mammalian models of experimental SCI, axons that traverse the lesion site frequently do so in close association with endogenous laminin deposits [12, 13]. Laminin has also been shown to enhance neuronal survival, modulate neuroinflammatory responses, and regulate the neural stem cell niche [14–16]

Polylaminin is a laboratory-engineered, stabilized polymer designed to reproduce key structural and functional features of native laminin [17]. In vitro, it enhances neurite outgrowth across multiple neuronal populations [18–20]. In a rat model of SCI, intramedullary administration shortly after injury promoted functional recovery, reduced inflammation and tissue loss, and supported axonal regeneration across the lesion site [21]. More recently, a veterinary study in dogs with chronic SCI reported that treatment with polylaminin, in combination with other agents, was well tolerated and associated with functional improvement [22].

In the present study, intramedullary delivery was selected to reproduce the route used in preclinical experiments in which regenerative effects were observed. Polylaminin forms supramolecular assemblies approximately 20 μm in length [23], and its biological activity depends on direct interaction with neuronal membranes and growth cones through integrin-mediated signaling and extracellular matrix–dependent mechanisms [18, 19]. Administration into the cerebrospinal fluid would likely result in dilution within the subarachnoid space and limited penetration across meningeal barriers into the injured parenchyma. Localized intramedullary deposition was therefore considered necessary to ensure direct contact with the lesion microenvironment and to reproduce the spatial conditions under which regenerative responses were demonstrated experimentally.

Given the absence of effective pharmacological therapies for acute SCI and the preclinical evidence supporting this approach, we conducted a first-in-human, investigator-initiated pilot study to evaluate the safety and feasibility of intramedullary polylaminin administration in patients with acute complete SCI. Neurological outcomes were explored as secondary endpoints. Although the study design does not allow conclusions regarding efficacy, it provides initial clinical data to inform the design of larger prospective studies.

## Methods

### Organization and design of the study

This was a prospective, investigator-initiated, multi-site, single-arm exploratory clinical trial designed to evaluate the safety and feasibility of an acute intramedullary injection of polylaminin at a dose of 1 μg/kg in individuals diagnosed with functionally complete spinal cord injury (SCI; AIS grade A). The study protocol was reviewed and approved by the Brazilian National Commission for Ethics in Research (CONEP; CAAE 3.474.884). The study was initiated in December 2016, prior to current requirements for prospective clinical trial registration, and retrospectively registered at the Brazilian Clinical Trials Registry (ReBEC), which is part of the World Health Organization International Clinical Trials Registry Platform (ICTRP-WHO). The ReBEC identifier code is RBR-9dfvgp, and the trial received the Universal Trial Number (UTN) U1111-1144-5390. This was an exploratory, non-sponsored academic study with no pre-established efficacy hypothesis.

A convenience sample of eight participants was included, reflecting the exploratory and first-in-human nature of the study. Because the primary objective was to assess feasibility and safety, rather than efficacy, no formal sample size calculation was performed.

Safety was evaluated through clinical examination, laboratory tests, and monitoring of medical complications. Neurological assessments were performed using the International Standards for Neurological Classification of Spinal Cord Injury (ISNCSCI), in association with the American Spinal Injury Association Impairment Scale (AIS), the most widely adopted system to standardize neurological outcomes in SCI individuals [3]. In this exploratory study, AIS grade progression was monitored to detect changes in neurological status. In addition, participants underwent imaging examinations, either magnetic resonance imaging (MRI; 5/8) or computed tomography (CT; 3/8), and, when feasible, electrophysiological testing including motor evoked potentials (MEP) and somatosensory evoked potentials (SSEP; 3/8) before treatment and at the end of the study.

**Fig. 1:**
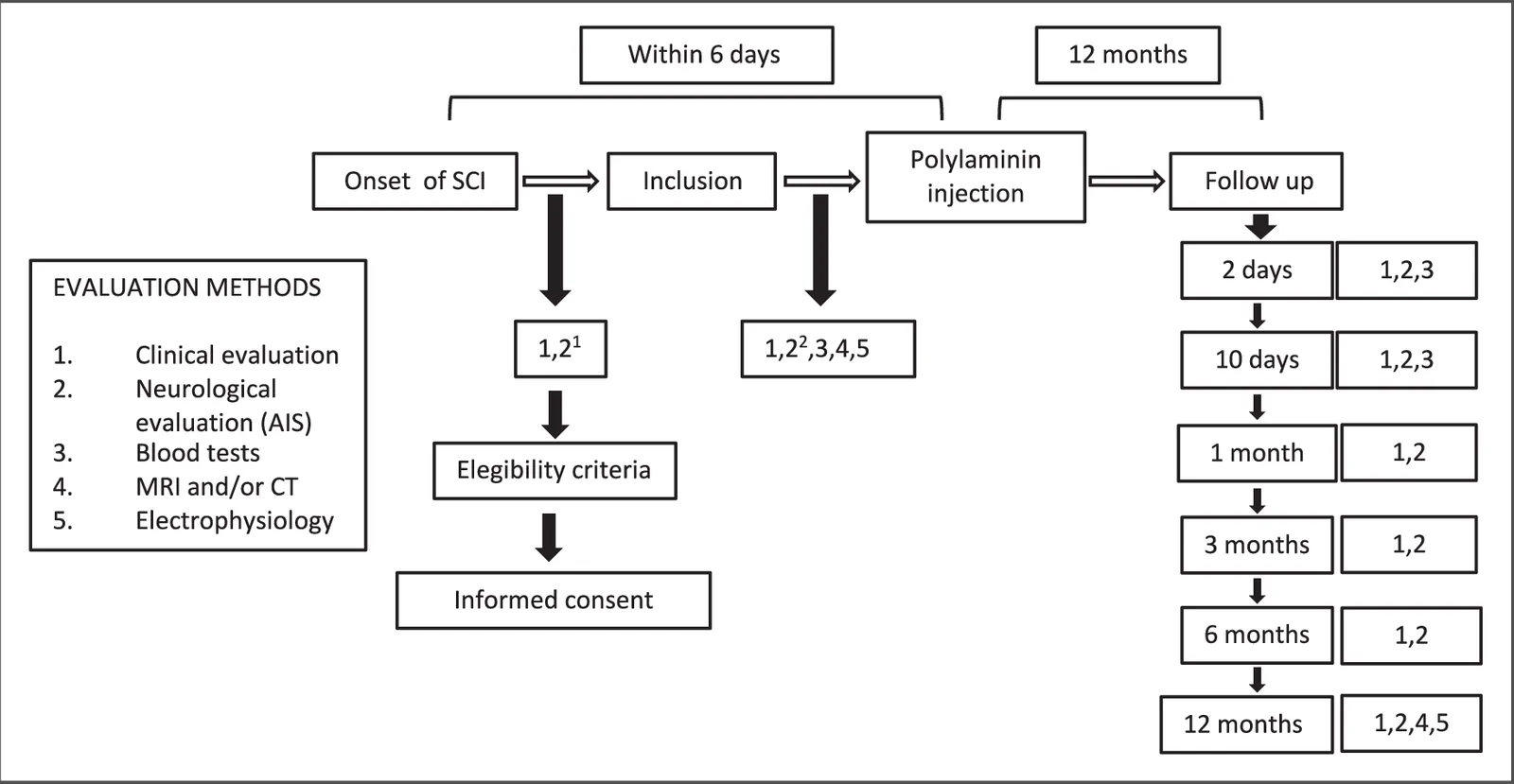
Flow chart of the study. ^1^The initial AIS assessment was performed by the on-call physician to determine eligibility for inclusion. ^2^The AIS grade was confirmed immediately before treatment by a study physician.

Participating hospitals are listed in Table S1. Inclusion criteria were age between 18 and 70 years and complete traumatic spinal cord injury (AIS A) with neurological level between C4 and T12. Exclusion criteria included severe traumatic brain injury, requirement for permanent mechanical ventilatory support, concomitant trauma considered by the study physicians to be severe enough to preclude neurological improvement, previous neurological disease, alcohol or illicit drug abuse or dependence, and any other comorbidity that, at the investigator’s discretion, would preclude inclusion in the study (Table S2). Follow-up visits were scheduled for days 2 ± 1, 10 ± 2, 30 ± 4, 90 ± 20, 180 ± 30, and 360 ± 30 after treatment. The study flow chart is shown in Fig. 1. After hospital discharge, participants were offered 200 h of neurofunctional physiotherapy, including neuromyofascial mobilization, proprioceptive neuromuscular facilitation (PNF/Kabat method), the Bobath concept, Pilates-based rehabilitation techniques, intensive functional training, and orthopedic manual therapy. Participants were also allowed to engage in additional rehabilitation programs of their choice.

### Participants

Written informed consent was obtained from all participants after the nature and possible consequences of the study had been explained. Participants depicted in the clinical photographs provided additional written consent for publication of these images. Facial features were obscured to protect participant privacy.

Only individuals with neurologically complete injuries (AIS grade A) were included. The initial neurological diagnosis was performed in two stages: a preliminary examination and a confirmatory examination.

The preliminary examination was conducted in the emergency setting by the on-call physician and included the assertive parameters of the AIS examination necessary to determine the presence or absence of sacral sparing, which defines a neurologically complete injury. These parameters included voluntary anal contraction (VAC), deep anal pressure (DAP), and light touch (LT) and pin-prick (PP) sensations in the sacral segments S4–S5. Absence of all four parameters indicates a complete injury (AIS grade A) [24].

A confirmatory examination was performed immediately before treatment administration by a neurosurgeon or physiatrist from the study team using the same four parameters, in order to increase diagnostic reliability while avoiding delays in clinically indicated interventions.

Follow-up examinations were performed using the complete ISNCSCI/AIS assessment by a trained physiatrist from the study team. All examinations were conducted with participants fully alert, oriented, and not under sedative medications. By the time of treatment administration, either the bulbocavernosus reflex or the Babinski sign was present in all participants, indicating resolution of spinal shock [25].

During hospitalization, patient care followed the standard clinical protocols of each participating hospital, except for the additional assessments and data collection required by the study. None of the participants received methylprednisolone.

### Study medication

Polylaminin at a final concentration of 100 μg/mL was prepared extemporaneously immediately before administration at the surgical site by mixing laminin (MilliporeSigma; Burlington, MA, USA; catalogue number CC085) with vehicle (20 mM acetate buffer containing 1 mM CaCl₂). The solution was transferred to a sterile wide-mouth vessel and drawn into a 1 mL syringe.

Polylaminin was delivered into the spinal cord through two intramedullary injections, each containing half of the total dose volume. Injections were performed in areas of preserved tissue immediately rostral and caudal to the lesion epicenter. Six participants required laminectomy for spinal cord decompression and received transdural intramedullary polylaminin injections during the surgical procedure. After laminectomy, a 26-gauge needle was inserted perpendicular to the spinal cord surface through the dura mater at the cranial and caudal margins of the lesion, targeting the posterior median sulcus under direct visualization. Injection depth was further confirmed by lateral fluoroscopic guidance, positioning the needle tip approximately halfway between the dorsal and ventral spinal cord surfaces on lateral projection prior to administration.

One participant had undergone decompression surgery prior to enrollment, and another did not require surgery because the transfixing gunshot injury produced no significant bone compression. In these two cases, polylaminin was administered percutaneously using 22-gauge spinal needles under fluoroscopic guidance with a C-arm image intensifier. The spinal midline was initially identified in the anteroposterior view, and the planned injection sites were marked using lateral fluoroscopy. Needle insertion was performed under lateral fluoroscopic visualization, following the trajectory and angulation previously determined from imaging studies and guided by the orientation of the spinous processes, until the interlaminar space was reached. Needle position was then verified in the anteroposterior projection to confirm alignment with the spinal midline before further advancement. Subsequently, under lateral fluoroscopic guidance, the needle was advanced into the spinal canal to a depth corresponding approximately to the midpoint between the lamina and the posterior wall of the vertebral body. The dura mater was punctured percutaneously, and no surgical durotomy was performed.

A human equivalent dose (HED) of 0.71 μg/kg was calculated based on the effective animal dose of 5 μg/kg previously used in female Wistar rats (200–250 g), assuming an average human body weight of 70 kg and applying standard allometric scaling according to FDA guidance for dose conversion [26].

$${{{\rm{HED}}}}={{{\rm{Animal\; dose}}}}\,\left({{{\rm{mg}}}}/{{{\rm{kg}}}}\right)\times {\left({{{\rm{animal\; weight}}}}\,/\,{{{\rm{human\; weight}}}}\right)}^{0.33}$$

This value was adjusted to 1 μg/kg based on the FDA definition of a microdose for exploratory clinical trials [27, 28]. According to this definition, a dose up to 100 μg (or 30 nM for protein products) is considered a microdose. In the present study, administered doses ranged from 55–80 μg (0.065–0.094 nM), as detailed in Table 1.

**Table 1:** Demography of the study.

Participant	Age	Sex	Cause	Injury level^a^	Date of injury	Time for treatment	Mass injected
1	32	F	Fall	C7	Dec 2016	1 day	70 μg
2	23	M	Motor vehicle accident	C7	Apr 2018	1 day	80 μg
3	31	M	Transfixing gunshot	C4	Sep 2018	2 days	80 μg
4	57	F	Fall	T2	Sep 2018	3 days	70 μg
5	30	M	Transfixing gunshot	T10	Oct 2018	1 day	70 μg
6	30	M	Motor vehicle accident	C5	Oct 2019	4 days	80 μg
7	41	F	Fall	T10	Sep 2020	6 days	55 μg
8	69	M	Fall after electrical shock	T4	May 2021	Less than 24 h	80 μg

^a^Neurological level of the injury.

### Statistical analysis

Given the exploratory nature of this first-in-human study and the small sample size, no formal hypothesis testing was performed. Data are presented using descriptive statistics.

Categorical variables, including AIS grade transitions, are reported as counts and proportions. Continuous variables are presented as ranges. Neurological outcomes were evaluated individually for each participant based on longitudinal ISNCSCI/AIS assessments performed during follow-up. Analyses focused on documenting safety and changes in neurological status over time.

## Results

### Demography of the Study

Eight participants were recruited between December 2016 and January 2021 and received treatment within 6 days after injury. The mean interval between trauma and polylaminin administration was 2.3 days. Four participants were treated within the first 24 h after trauma, whereas the remaining four received treatment at 2, 3, 4, and 6 days post-injury. Demographic characteristics are summarized in Table 1.

The cohort included five males and three females (62.5% male), aged 23–69 years (mean 39.1). Causes of injury were falls (*n* = 4), motor vehicle accidents (*n* = 2), and transfixing gunshot wounds (*n* = 2). Neurological injury levels ranged from C5 to T10, with four participants presenting tetraplegia and four paraplegia.

Seven of the eight participants underwent early decompression and stabilization surgery according to clinical indication. Participant 3 had no surgical indication and received treatment percutaneously. Participant 6 also received a percutaneous injection because study enrollment occurred after surgical stabilization had already been completed. Representative frames from the initial imaging examinations are shown in Figure S1.

### Medical complications

All medical complications occurring from the time of inclusion until the end of hospitalization were recorded in the study database and treated according to standard clinical practice. After completion of the trial, complications were independently reviewed by an external physician (GSH) and classified according to the system proposed by the North American Clinical Trials Network for Spinal Cord Injury [29]. These events are summarized in Table 2.

**Table 2:** Medical complications classified by organ system and severity.

Type of complication	Total number of complications				No. of participants
	*severe*	*moderate*	*mild*	total	
Infectious	8	5	1	14	7
GI/GU	4	3	8	15	7
Neuropsychiatric	3	5	6	14	7
Skin	1	3	1	4	4
Cardiovascular	8	1	1	10	3
Pulmonary	6	3	0	9	3
Hematological	2	0	0	2	2
Others^a^	0	1	1	2	2
Total	32	21	18	71	
% Total	45	30	25	100	

^a^Includes one case of lower limb oedema (moderate) and one case of mispositioned arthrodesis hardware (mild).

All participants presented at least one medical complication during hospitalization. Infectious events were the most frequent, occurring in seven participants within the first month after injury. Eight of the fourteen infection-related complications were classified as severe. The second and third most frequent complication categories were gastrointestinal/genitourinary (GI/GU) and neuropsychiatric events, also affecting seven participants but generally with lower severity. Four participants developed skin complications, mostly pressure ulcers. Cardiovascular and pulmonary complications occurred in three participants, while hematological events were observed in two. Severe complications occurring within the first 30 days are detailed in Table S3. Considering only severe events, infections remained the most common complication, affecting 5 of 8 participants (63%). Pulmonary and GI/GU complications each occurred in three participants (38%). Cardiovascular, neurological/psychiatric, and hematological complications were observed in two participants (25%), whereas severe skin ulcers during hospitalization occurred in one participant (17%). A comprehensive list of complications is provided in Table S4.

Three participants died during the course of the study. Participant 1 presented signs of broncho aspiration at admission and developed severe pneumonia, leading to death five days after treatment. Participant 3 experienced a sudden cardiovascular event on day 20 of follow-up, with pericardial effusion and cardiac tamponade, likely related to the initial traumatic injury. Participant 8 was discharged after three weeks of hospitalization and completed the one-month follow-up at home during the COVID-19 pandemic but was later hospitalized due to an infected pressure ulcer and died from sepsis two months after inclusion.

All deaths were reported to the Brazilian national ethics authority, National Commission for Ethics in Research, which authorized continuation of the study after determining that the events could not be attributed to the experimental intervention.

### Monitoring of hepatic and renal markers of toxicity

Blood tests were performed before treatment and compared with measurements obtained 2 and 10 days after treatment to monitor potential renal and hepatic toxicity (Table S5). Creatinine and urea were assessed in all participants. The only deviations consisted of mild elevations ( < 1.2-fold above reference values) observed for creatinine in participant 8, already present before treatment and persisting across the three assessments, and a mild elevation in urea in participant 6 at the two-day evaluation. For alanine and aspartate aminotransferases (ALT and AST), most deviations were classified as mild. Two moderate elevations were detected in participants 2 and 8 but were already present prior to treatment and normalized by the 10-day evaluation. No severe elevations of liver enzymes were observed. No clinically relevant abnormalities were detected in blood counts, apart from mild anemia in the immediate postoperative period in some participants (Table S6).

### Neurological evaluation

Neurological outcomes were evaluated as exploratory endpoints in this pilot safety study. The evolution of neurological status according to the ASIA Impairment Scale (AIS) is summarized in Table 3.

**Table 3:** Neurological evaluation.

Participant		Before injection	2 dpi	10 dpi	1 mpi	3 mpi	6 mpi	12 mpi
1	DAP	No						
	VAC	No						
	AIS	A 24 hpt						
2	DAP	No	No	Yes	Yes	Yes	Yes	Yes
	VAC	No	No	No	Yes	Yes	Yes	Yes
	AIS	A 24 hpt	A	B	D	D	D	D
3	DAP	No	Yes	NT				
	VAC	No	No	No				
	AIS	A 36 hpt	C^a^	ND				
4	DAP	No	Yes	No	No	No	Yes	No
	VAC	No	No	No	No	Yes	No	Yes
	AIS	A 72 hpt	ND^b^	A	A	C	C^a^	C
5	DAP	No	No	No	No	No	No	Yes
	VAC	No	No	No	No	Yes	Yes	Yes
	AIS	A 24 hpt	A	A	A	C	C	C
6	DAP	No	No	Yes	Yes	Yes	Yes	Yes
	VAC	No	No	No	No	Yes	No	Yes
	AIS	A 96 hpt	ND^c^	B	B	C	B^d^	C
7	DAP	No	No	No	No	Yes	Yes	Yes
	VAC	No	No	No	Yes	Yes	Yes	No
	AIS	A 144 hpt	A	A	C	C	C	C^a^
8	DAP	No	No	No	No			
	VAC	No	No	No	Yes			
	AIS	A 24 hpt	A	A	C^e^			

^a^DAP preserved with some sparing of motor function more than three levels below the ipsilateral motor level.

^b^PP and LT could not be assessed in the cervical region due to collar immobilization.

^c^Participant sedated.

^d^No VAC detected; however, the participant reported pain due to an anal fissure.

^e^DAP preserved with some sparing of motor function more than three levels below the ipsilateral motor level, with contraction of non-key muscles at L4. Participants 1 and 3 died during hospitalization, and participant 8 died after discharge.

*dpi* days post-injection, *mpi* months post-injection, *hpt* hours post-trauma, *ND* not determined, *NT* not testable, *DAP* deep anal pressure, *VAC* voluntary anal contraction.

Participant 1 could not be evaluated after treatment administration due to early clinical deterioration requiring sedation and mechanical ventilation, and therefore no post-treatment neurological assessment was available. Participant 3 showed early neurological improvement within 48 h after treatment, which may reflect either early recovery or limitations of initial classification at very early time points.

Three participants (2, 7, and 8) regained voluntary motor control, converting to at least AIS C by the one-month examination. Three additional participants (4, 5, and 6) converted to AIS C after three months of follow-up. Overall, six of eight treated participants converted from AIS A to AIS C or D during follow-up.

Importantly, in all six participants who demonstrated AIS conversion, the initial diagnosis of complete injury (AIS A) was considered reliable based on timing of assessment. In three cases (participants 4, 6, and 7), the first neurological examination was performed 72 h or more after injury [30]. In the remaining three cases (participants 2, 5, and 8), AIS A classification was confirmed at a 48 h reassessment. Thus, all participants contributing to the observed neurological improvement had confirmation of complete injury beyond the earliest acute phase.

Among the six participants who survived the hospitalization period, voluntary motor function was observed during follow-up in all individuals. Although the small sample size precludes formal statistical comparison, the observed 75% conversion rate (6/8) appears higher than rates reported in observational cohorts of AIS A individuals.

**Fig. 2:**
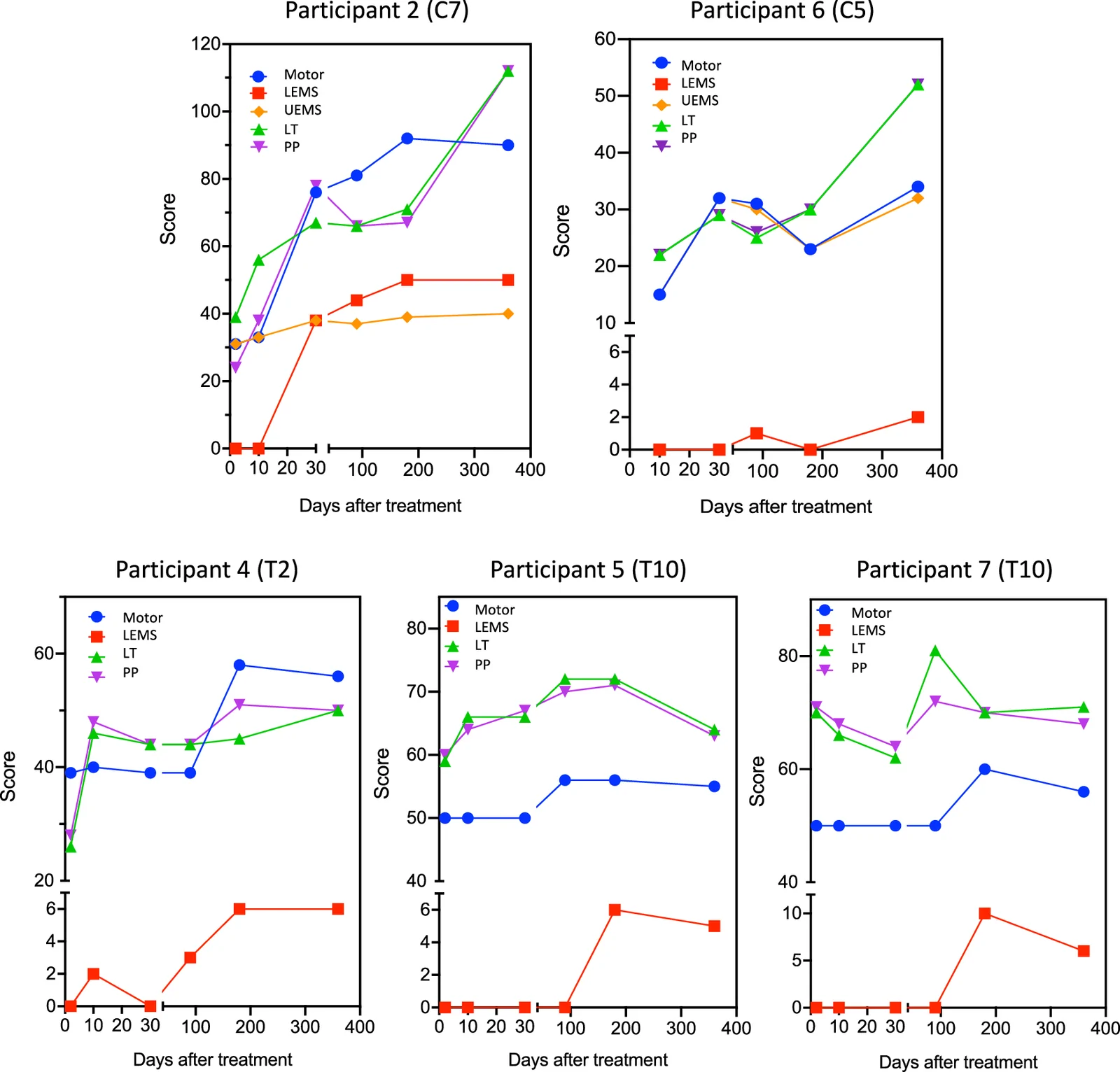
Motor and sensory scores. Total motor scores (Motor), lower extremity motor scores (LEMS), upper extremity motor scores (UEMS), light touch (LT), and pin prick (PP) sensitivities were assessed at 2, 10, 30, 90, 180, and 360 days after treatment. Graphs are grouped according to lesion level: cervical (upper panels) and thoracic (lower panels). Note that different y-axis scales were used to highlight the observed changes.

**Fig. 3:**
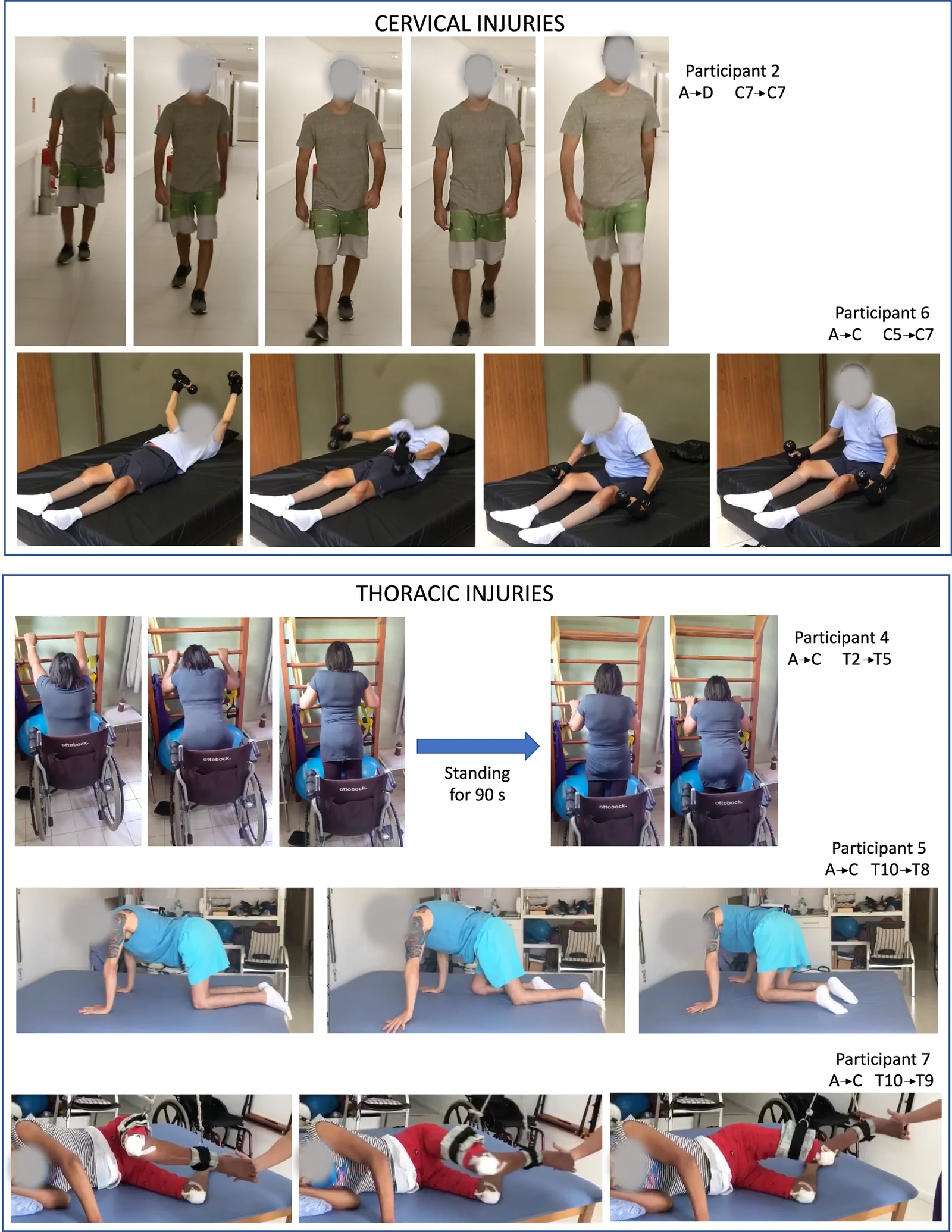
Motor activity. Representative frames illustrating motor activities performed by each participant at the end of follow-up. Among participants with cervical injuries, participant 2 (C7 at admission) achieved independent overground walking, and participant 6 (C5 at admission) demonstrated sufficient trunk stability to sit without assistance. Among thoracic participants, participant 4 (T2 at admission) was able to stand, maintain the standing position for more than one minute, and sit down without orthosis, using a support bar for stabilization. Participant 5 (T10, transfixing injury) was able to crawl, and participant 7 (T10) demonstrated leg movement in a gravity-eliminated condition.

Motor and sensory scores for the five participants completing the 12-month follow-up are shown in Fig. 2. Recovery of both motor and sensory scores was more pronounced among participants with cervical injuries. Participant 2 recovered normal sensory function across all dermatomes, regained independent bladder and bowel control, and achieved full overground walking capacity (Fig. 3). Participant 6 improved his neurological level from C5 to C7 by the final evaluation. In addition to recovery of voluntary anal contraction (VAC) and deep anal pressure (DAP), he regained bilateral sensation at S3–S5, became able to localize tactile stimuli on the muscles of both legs (although not on the skin), and developed voluntary contractions in muscles innervated by C8, T1, L2, and S1 on the left side. Throughout follow-up, voluntary contractions were observed in alternating key and non-key muscles (Fig. 3).

Among paraplegic participants, sensory recovery beyond DAP detection was limited (Fig. 2). Improvements in lower extremity motor scores (LEMS) began at three months for participant 4 and at six months for participants 5 and 7. Gains of 6–10 LEMS points were documented for these individuals, with a tendency toward stabilization or slight reduction at the 12-month evaluation.

### Imaging

**Fig. 4:**
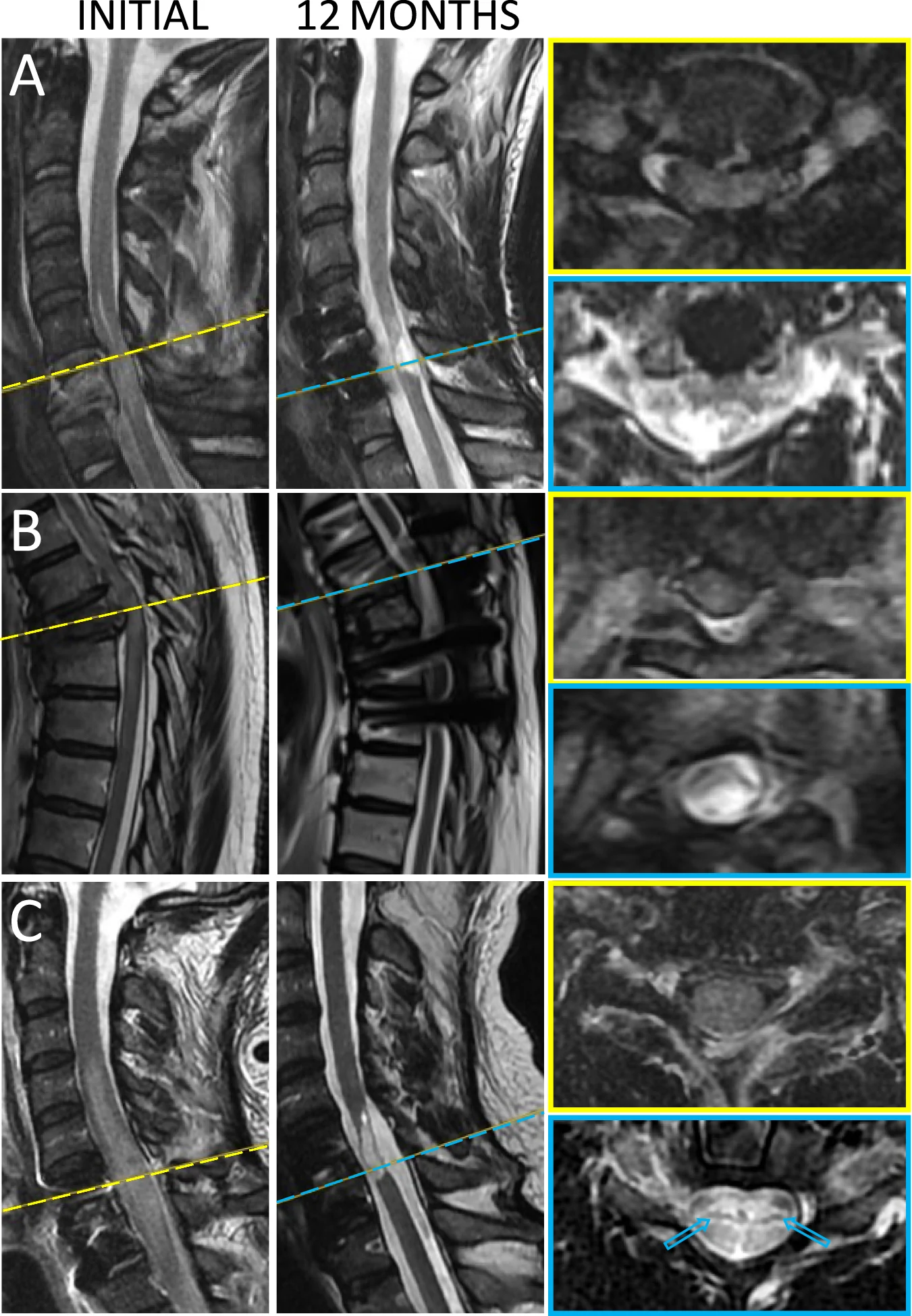
Initial and final MRI images. **A** T2-weighted sagittal and axial sequences of participant 2 obtained before and 12 months after polylaminin injection. Initial images show a fractured C6 vertebra and extensive oedema throughout the spinal cord cross-section. At 12 months, an oval-shaped hyperintense area consistent with fluid occupied the lesion site. **B** T2-weighted sagittal and axial sequences of participant 4 before and 12 months after treatment. Final MRI images were affected by prosthesis-related artifacts; however, findings suggest the formation of a cystic cavity at the lesion site. **C** T2-weighted sagittal and axial MRI sequences of participant 6 before and at 12 months after treatment. Initial images reveal extensive oedema across the entire spinal cord cross-section, with interruption of cerebrospinal fluid flow. Final images show marked hyperintensity at the lesion site, adjacent to a narrow strip of preserved tissue (arrows in axial view).

Initial and final imaging examinations were compared for the five participants who completed the 12-month follow-up. Participant 2 developed a large cystic cavity within the spinal cord occupying most of its cross-section (Fig. 4A). Participant 4 also developed a cavity at the injury site (Fig. 4B). At 12 months, participant 6 presented only a narrow strip of preserved tissue at the center of the spinal cord (Fig. 4C). Participant 5 did not undergo MRI because of metallic projectile fragments. CT comparison did not reveal notable changes during follow-up. Participant 7 had only the final MRI examination performed because MRI was unavailable at the hospitalization center and transfer was not feasible during the peak of the COVID-19 pandemic. Overall, imaging findings were consistent with the expected evolution after spinal cord injury and did not reveal radiological changes attributable to the treatment.

### Electrophysiological assessment

**Fig. 5:**
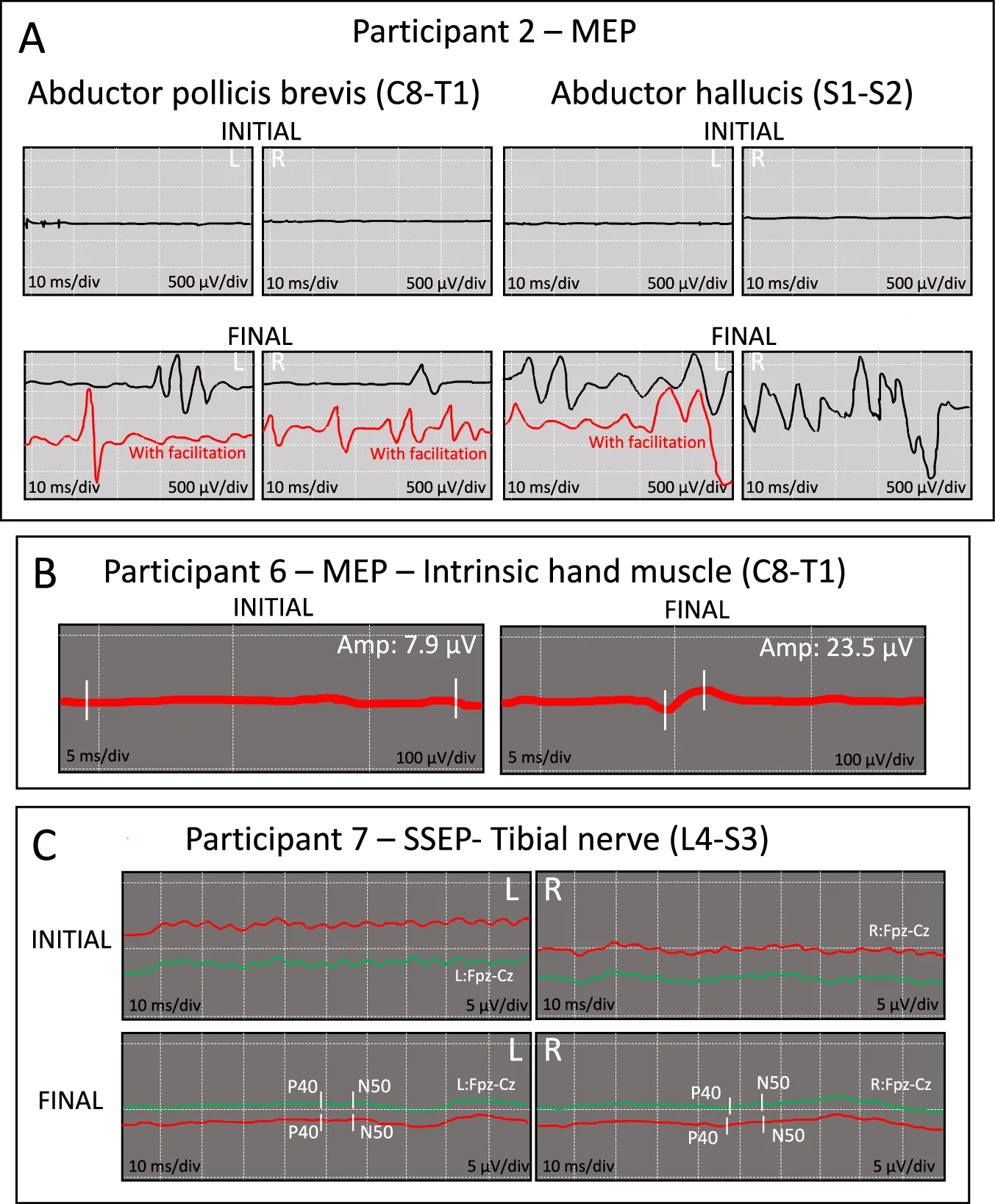
Electrophysiological assessment. **A** Motor evoked potentials (MEPs) of participant 2 recorded using Neurosoft equipment with transcranial electrical stimulation at C3 and C4 (10–20 system) and recordings from abductor pollicis brevis and abductor hallucis muscles. Final MEPs were obtained using transcranial magnetic stimulation (Neuro-MEP-Micro system). **B** MEPs of participant 6 recorded with a Medtronic NIM Eclipse (32-channel system), using transcranial electrical stimulation at C3 and C4 (10–20 system), with recordings from intrinsic hand muscles of the right upper limb. **C** Somatosensory evoked potentials (SSEPs) of participant 7 obtained by stimulation of the tibial nerves at the ankles (P40/N50), with recordings at CZ′–FPZ and FPZ–C3/FPZ–C4 (10–20 system). L and R indicate left and right, respectively.

Electrophysiological examinations were performed in participants 2, 6, and 7 using motor evoked potentials (MEP) and somatosensory evoked potentials (SSEP). Participant 2 initially presented SSEP patency only in the right upper limb, whereas MEP responses were present only down to C7, with no detectable responses at C8–T1 or in the lower limbs (Fig. 5A). At the final examination, MEP responses reappeared in distal hand muscles, particularly on the left side, although with delayed latency. Motor potentials were also detected in the adductor hallucis (S1–S2) despite delayed conduction. Final SSEP recordings showed abnormal but detectable responses in both median and tibial nerves.

Participant 6 initially showed normal SSEP conduction in the median nerves and reduced amplitudes in tibial nerve responses. MEP responses were present down to C6–C7 and absent at C8–T1 and in the lower limbs. At the final examination, SSEP findings remained unchanged, but MEP revealed a previously absent response at C8–T1 in the right hand (Fig. 5B).

Participant 7 presented normal SSEP and MEP responses in the upper limbs throughout follow-up. Initial SSEP evaluation showed absence of responses in the lower limbs, whereas the final examination revealed low-amplitude potentials in tibial nerves identified by P40/N50 markers (Fig. 5C). MEP findings remained unchanged.

## Discussion

The primary objective of this trial was to evaluate the feasibility and safety of intramedullary polylaminin injection in individuals with acute traumatic spinal cord injury. The procedure was generally well tolerated, with no findings suggesting major safety concerns associated with the intervention. Although the study was not designed to assess efficacy, conversion from AIS A to C or D was observed at a frequency higher than typically reported in the natural history of comparable injuries.

The adverse events observed in the present cohort were qualitatively similar to those commonly reported in patients with acute spinal cord injury [29]. Individuals with SCI frequently develop dysautonomia and cardiopulmonary dysfunction, conditions that profoundly affect systemic physiology [31]. All participants in the present study experienced at least one medical complication during hospitalization. Although this represents a high prevalence, it is comparable to previously reported complication rates of 63–84% among AIS A patients in large clinical registries [29, 32].

Three deaths occurred among the eight participants, caused by pneumonia, pericardial effusion with cardiac tamponade, and sepsis. The first two deaths occurred during hospitalization, whereas the third occurred one month after discharge. Hemodynamic instability due to loss of vasoconstrictor tone, together with respiratory impairment caused by diaphragmatic and intercostal muscle dysfunction, predisposes SCI patients to cardiovascular complications and pneumonia, the latter being a leading cause of mortality in this population. Pneumonia and sepsis (participants 1 and 8, respectively) could in principle be related to a sustained anti-inflammatory state potentially induced by the treatment, as polylaminin has been reported to display anti-inflammatory properties in experimental models [21]. However, clinical laboratory records did not reveal leukopenia or abnormally reduced levels of C-reactive protein during the postoperative period, findings that argue against a systemic immunosuppressive effect. In all three cases, detailed analysis of the clinical course suggested that the fatal outcomes were related to the underlying medical condition rather than to the experimental intervention.

Mortality rates reported for acute SCI vary widely across clinical settings. A recent study conducted in emergency centers in Japan reported that 43.8% of cervical SCI patients classified as AIS A died during hospitalization [33]. Similarly, a Brazilian retrospective study reported mortality rates of approximately 46% among patients with cervical injuries [34]. Another investigation conducted between 1996 and 2011 in public hospitals in Rio de Janeiro, similar to the clinical setting of the present study, found mortality rates ranging from less than 5% to more than 40%, depending on the year and on whether the hospital was managed by state or municipal health authorities [35].

Across large observational studies, conversion from AIS A to motor-incomplete grades (AIS C or D) typically ranges from 0 to approximately 15% [3, 4, 36, 37]. In the present study we found that, among the 8 participants, all initially diagnosed as AIS A, 5 converted to C and 1 to AIS D. Importantly, all these six participants had confirmation of complete injury (AIS A) beyond the earliest acute phase. In three cases, the first neurological assessment was performed at or beyond 72 h after injury, whereas in the remaining cases AIS A classification was confirmed at a 48 h reassessment, in conjunction with repeated neurological examination including assessment of sacral sparing and reflexes indicative of resolution of spinal shock. This reduces the likelihood that early neurological improvement reflects misclassification during spinal shock, a recognized limitation in acute SCI studies.

It is important to note that the cohort in the present study was highly heterogeneous, encompassing both cervical and thoracic injuries, as well as contusive non-penetrating trauma and gunshot injuries, a feature that imposes limitations on the interpretation of the results. Considering only thoracic injuries, 4 out of 4 participants converted from AIS A to C. Among the four participants with cervical injuries, two died during hospitalization, whereas the remaining two converted to AIS C and D. There were two cases of transfixing gunshot injuries in the cohort, one cervical and one thoracic. Although the pathophysiology of penetrating injuries differs substantially from that of non-penetrating trauma, the decision not to exclude such cases was mainly based on the proposed mechanism of action of polylaminin, namely the promotion of axonal regeneration independently of the mechanism of injury [21]. In this regard, our previous study demonstrating axonal regeneration induced by polylaminin in rats included a model of complete spinal cord transection, in which neuronal cell bodies were retrogradely labeled at the cranial stump after tracer injection caudal to the lesion site. In the same study, polylaminin was also shown to be effective after dorsal hemisection and moderate balloon compression injuries [21]. If participants with penetrating injuries were excluded from the analysis, 5 out of 6 participants would still have converted from AIS A to C or D.

Recent years have seen a growing interest in the use of bioengineered scaffolds for spinal cord injury, aiming not only to bridge cavities and provide structural support for regeneration, but also to deliver signaling molecules capable of modulating regeneration and tissue repair [38]. Interestingly, many currently investigated clinical scaffolds are primarily based on collagen I or fibrin matrices, extracellular matrix elements that are not classically associated with axonal growth to the same extent as laminin during nervous system development and peripheral nerve regeneration [39]. In this context, polylaminin may itself be regarded as a signaling scaffold, since it combines the structural properties of a polymerized biomaterial with the presentation of native laminin binding sites. Rather than introducing growth factors or synthetic signaling motifs into biomaterials with limited intrinsic association with axonal growth, the present approach was based on reproducing the biologically polymerized state of laminin in a biomimetic manner [17]. In line with this concept, laminin-derived signaling domains have been associated with some of the most substantial regenerative effects reported in the scaffold literature. These include peptide amphiphiles functionalized with laminin epitopes, which promoted robust axonal regeneration and functional recovery in preclinical models [40], as well as an exploratory clinical case series combining laminin/chitosan scaffolds with mesenchymal stem cells in chronic spinal cord injury [41].

The observation that a single administration of polylaminin was temporally associated with changes in AIS classification in a subset of participants raises the possibility that even transient exposure of the injured spinal cord to laminin-based matrices may influence the regenerative microenvironment. Experimental studies have previously shown that brief contact with laminin can markedly accelerate axonal outgrowth in cultured sensory neurons [42]. Alternatively, the injected protein, particularly in its polymerized form, may persist within the injured spinal cord for sufficient time to support axonal elongation and structural remodeling. An intriguing aspect of the present findings is that neurological improvement occurred in the absence of clear imaging evidence of large-scale tissue regeneration. This observation does not exclude the possibility that relatively small numbers of regenerating axons could contribute to functional recovery. Even limited reconnection of descending pathways may be sufficient to provide input to intrinsic spinal central pattern generator networks, which can coordinate rhythmic motor activity [43, 44]. Previous studies have demonstrated that hierarchical integration of local central pattern generators can synchronize segmental networks responsible for locomotion, posture, and autonomic control [45]. In this context, restoration of a small number of descending serotonergic or adrenergic projections might allow supraspinal modulation of spinal circuitry, in a manner conceptually similar to the rhythmic motor patterns that can be induced in paralyzed individuals by epidural electrical stimulation of the spinal cord [46].

Several limitations should be acknowledged. This was a small exploratory pilot study involving only eight participants, and the absence of a control group precludes any causal inference regarding treatment efficacy. In addition, neurological recovery after acute traumatic spinal cord injury is known to display substantial interindividual variability, and spontaneous improvement cannot be entirely excluded as a contributing factor. Although participants engaged in structured and complementary rehabilitation programs during follow-up, such interventions are not typically associated with recovery of voluntary motor function in individuals initially classified as AIS A [47]. The timing between injury and treatment also varied among participants, reflecting the logistical constraints inherent to emergency clinical care. Furthermore, the study was not designed or powered to formally assess efficacy outcomes. These factors limit the generalizability of the present findings and highlight the need for larger clinical studies to more rigorously evaluate the safety profile and potential therapeutic effects of polylaminin in human spinal cord injury.

In conclusion, a single low-dose intramedullary administration of polylaminin in the acute phase of traumatic spinal cord injury appeared to be uneventful and was not associated with unexpected safety concerns in this small pilot cohort. The neurological improvements observed during follow-up warrant further investigation in larger clinical studies designed to evaluate the potential therapeutic role of polylaminin in human spinal cord injury.

## Supplementary information

Supplementary files

## Data Availability

Data are securely stored in a REDCap database and may be made available upon reasonable request to the corresponding author, in accordance with institutional and ethical guidelines. De-identified individual participant data are not currently available in a public repository.

## References

[1] Ahuja CS, Nori S, Tetreault L, Wilson J, Kwon B, Harrop J, et al. Traumatic spinal cord injury-repair and regeneration. Neurosurgery. 2017;80:S9–S22. 10.1093/neuros/nyw080 28350947

[2] Griffin M, Bradke F. Therapeutic repair for spinal cord injury: combinatory approaches to address a multifaceted problem. EMBO Mol Med. 2020;12:e11505. 10.15252/emmm.201911505 PMC7059014 32090481

[3] Kirshblum S, Snider B, Eren F, Guest J. Characterizing natural recovery after traumatic spinal cord injury. J Neurotrauma. 2021;38:1267–84. 10.1089/neu.2020.7473 PMC8080912 33339474

[4] Aimetti AA, Kirshblum S, Curt A, Mobley J, Grossman RG, Guest JD. Natural history of neurological improvement following complete (AIS A) thoracic spinal cord injury across three registries to guide acute clinical trial design and interpretation. Spinal Cord. 2019;57:753–62. 10.1038/s41393-019-0299-8 PMC6760562 31182786

[5] Khorasanizadeh M, Yousefifard M, Eskian M, Lu Y, Chalangari M, Harrop JS, et al. Neurological recovery following traumatic spinal cord injury: a systematic review and meta-analysis. J Neurosurg Spine. 2019;30:683–99. 10.3171/2018.10.SPINE18802 30771786

[6] Barros CS, Franco SJ, Müller U. Extracellular matrix: functions in the nervous system. Cold Spring Harb Perspect Biol. 2011;3:a005108. 10.1101/cshperspect.a005108 PMC3003458 21123393

[7] Miner JH, Yurchenco PD. Laminin functions in tissue morphogenesis. Annu Rev Cell Dev Biol. 2004;20:255–84. 10.1146/annurev.cellbio.20.010403.094555 15473841

[8] Chen ZL, Haegeli V, Yu H, Strickland S. Cortical deficiency of laminin gamma1 impairs the AKT/GSK-3β signaling pathway and leads to defects in neurite outgrowth and neuronal migration. Dev Biol. 2009;327:158–68. 10.1016/j.ydbio.2008.12.006 PMC2669444 19118544

[9] Kang M, Yao Y. Laminin regulates oligodendrocyte development and myelination. Glia. 2022;70:414–29. 10.1002/glia.24117 PMC8817735 34773273

[10] Liesi P. Do neurons in the vertebrate CNS migrate on laminin? EMBO J. 1985;4:1163–70. 10.1002/j.1460-2075.1985.tb03755.x PMC554319 4006911

[11] Chen ZL, Strickland S. Laminin gamma1 is critical for Schwann cell differentiation, axon myelination, and regeneration in the peripheral nerve. J Cell Biol. 2003;163:889–99. 10.1083/jcb.200307068 PMC2173689 14638863

[12] Grimpe B, Dong S, Doller C, Temple K, Malouf AT, Silver J. The critical role of basement membrane-independent laminin gamma 1 chain during axon regeneration in the CNS. J Neurosci. 2002;22:3144–60. 10.1523/JNEUROSCI.22-08-03144.2002 PMC6757543 11943817

[13] Anderson MA, O’Shea TM, Burda JE, Ao Y, Barlatey SL, Bernstein AM, et al. Required growth facilitators propel axon regeneration across complete spinal cord injury. Nature. 2018;561:396–400. 10.1038/s41586-018-0467-6 PMC6151128 30158698

[14] Zhang D, Yang S, Toledo EM, Gyllborg D, Saltó C, Villaescusa JC, et al. Niche-derived laminin-511 promotes midbrain dopaminergic neuron survival and differentiation through YAP. Sci Signal. 2017;10:eaa14165. 10.1126/scisignal.aal4165 28831020

[15] Hallmann R, Hannocks MJ, Song J, Zhang X, Di Russo J, Luik AL, et al. The role of basement membrane laminins in vascular function. Int J Biochem Cell Biol. 2020;127:105823. 10.1016/j.biocel.2020.105823 32781135

[16] Nascimento MA, Sorokin LM, Coelho-Sampaio T. Fractone bulbs derive from ependymal cells and their laminin composition influence the stem cell niche in the subventricular zone. J Neurosci. 2018;38:3880–9. 10.1523/JNEUROSCI.3064-17.2018 PMC6705924 29530987

[17] Barroso MM, Freire E, Limaverde GSC, Rocha GM, Batista EJO, Weissmüller G, et al. Artificial laminin polymers assembled in acidic pH mimic basement membrane organization. J Biol Chem. 2008;283:11714–20. 10.1074/jbc.M709301200 18276595

[18] Freire E, Gomes FCA, Linden R, Moura Neto V, Coelho-Sampaio T. Structure of laminin substrate modulates cellular signaling for neuritogenesis. J Cell Sci. 2002;115:4867–76. 10.1242/jcs.00173 12432074

[19] Hochmann-Mendez C, Menezes JRL, Sholl-Franco A, Coelho-Sampaio T. Polylaminin recognition by retinal cells. J Neurosci Res. 2014;92:24–34. 10.1002/jnr.23298 24265135

[20] Haggerty AE, Al-Ali H, Oudega M. Soluble laminin polymers enhance axon growth of primary neurons in vitro. J Biomed Mater Res A. 2018;106:2372–81. 10.1002/jbm.a.36429 29637694

[21] Menezes K, Menezes JRL, Nascimento MA, Santos RS, Coelho-Sampaio T. Polylaminin, a polymeric form of laminin, promotes regeneration after spinal cord injury. FASEB J. 2010;24:4513–22. 10.1096/fj.10-157628 20643907

[22] Chize CM, Vivas DG, Menezes K, Freire M, Jiddu RFP, Graça-Souza AV, et al. A laminin-based therapy for dogs with chronic spinal cord injury: promising results of a longitudinal trial. Front Vet Sci. 2025;12:1592687. 10.3389/fvets.2025.1592687 PMC12380836 40881640

[23] Hochmann-Mendez C, Cantini M, Moratal D, Salmeron-Sanchez M, Coelho-Sampaio T. A fractal nature for polymerized laminin. PLoS One. 2014;9:e109388. 10.1371/journal.pone.0109388 PMC4190072 25296244

[24] Rupp F, Biering-Sørensen F, Burns SP, Graves DE, Guest J, Jones L, et al. International standards for neurological classification of spinal cord injury: Revised 2019. Top Spinal Cord Inj Rehabil. 2021;27:1–22. 10.46292/sci2702-1 PMC8152171 34108832

[25] Ditunno JF, Little JW, Tessler A, Burns AS. Spinal shock revisited: a four-phase model. Spinal Cord. 2004;42:383–95. 10.1038/sj.sc.3101603 15037862

[26] FDA. Guidance for Industry: Estimating the Maximum Safe Starting Dose in Initial Clinical Trials for Therapeutics in Adult Healthy Volunteers. Rockville, MD, USA: FDA; 2005.

[27] FDA. Guidance for Industry, Investigators, and Reviewers: Exploratory IND Studies. Rockville, MD: Food and Drug Administration; 2006.

[28] Burt T, Young G, Lee W, Kusuhara H, Langer O, Rowland M, et al. Phase 0/microdosing approaches: time for mainstream application in drug development? Nat Rev Drug Discov. 2020;19:801–18. 10.1038/s41573-020-0080-x 32901140

[29] Grossman RG, Frankowski RF, Burau KD, Toups EG, Crommett JW, Johnson MM, et al. Incidence and severity of acute complications after spinal cord injury. J Neurosurg Spine. 2012;17:119–28. 10.3171/2012.5.AOSPINE12127 22985378

[30] Scivoletto G, Di Donna V. Prediction of walking recovery after spinal cord injury. Brain Res Bull. 2009;78:43–51. 10.1016/j.brainresbull.2008.06.002 18639616

[31] Ball PA. Critical care of spinal cord injury. Spine. 2001;26:S27–S30. 10.1097/00007632-200112151-00006 11805605

[32] Jiang F, Jaja BNR, Kurpad SN, Badhiwala JH, Aarabi B, Grossman RG, et al. Acute adverse events after spinal cord injury and their relationship to long-term neurologic and functional outcomes: analysis from the North American clinical trials network for spinal cord injury. Crit Care Med. 2019;47:e854–e862. 10.1097/CCM.0000000000003937 31389834

[33] Higashi T, Eguchi H, Wakayama Y, Sumi M, Saito T. Risk factors associated with mortality after traumatic cervical spinal cord injury. OTA Int. 2018;1:e003. 10.1097/OI9.0000000000000003 PMC7953704 33937641

[34] Neumann CR, Brasil AV, Albers F. Risk factors for mortality in traumatic cervical spinal cord injury: Brazilian data. J Trauma. 2009;67:67–70. 10.1097/TA.0b013e3181aa63f3 19590310

[35] Santos TSC, Guimarães RM, Boeira SF. Spinal cord injury epidemiology in public emergency rooms in the municipality of rio de janeiro. Esc Anna Nery. 2012;16:747–53.

[36] Burns AS, Lee BS, Ditunno JF Jr, Tessler A. Patient selection for clinical trials: the reliability of the early spinal cord injury examination. J Neurotrauma. 2003;20:477–82. 10.1089/089771503765355540 12803979

[37] Scivoletto G, Tamburella F, Laurenza L, Torre M, Molinari M. Who is going to walk? A review of the factors influencing walking recovery after spinal cord injury. Front Hum Neurosci. 2014;8:141. 10.3389/fnhum.2014.00141 PMC3952432 24659962

[38] Khavandegar A, Dehnavi NS, Ganau M, Ramezani Z, Khodadoust E, Tabatabaei MSHZ, et al. Neuroregenerative and neuroprotective effects of bioengineered scaffolds in spinal cord injury: a systematic review of preclinical and early phase clinical studies. Spinal Cord. 2025;63:451–69. 10.1038/s41393-025-01114-9 40849360

[39] Santiago-Medina MyersJP, Gomez M. TM. Regulation of axonal pathfinding by integrin-ECM interactions. Dev Neurobiol. 2011;71:901–23. 10.1002/dneu.20931 PMC3192254 21714101

[40] Álvarez Z, Kolberg-Edelbrock AN, Sasselli IR, Ortega JE, Qiu R, Syrgiannis Z, et al. Bioactive scaffolds with enhanced supramolecular motion promote recovery from spinal cord injury. Science. 2021;274:848–56. 10.1126/science.abh3602 PMC8723833 34762454

[41] Amr SM, Gouda A, Koptan WT, Galal AA, Abdel-Fattah DS, Rashed LA, et al. Bridging defects in chronic spinal cord injury using peripheral nerve grafts combined with a chitosan-laminin scaffold and enhancing regeneration through them by co-transplantation with bone-marrow-derived mesenchymal stem cells: case series of 14 patients. J Spinal Cord Med. 2014;37:54–71. 10.1179/2045772312Y.0000000069 PMC4066552 24090088

[42] Kuhn TB, Schmidt MF, Kater SB. Laminin and fibronectin guideposts signal sustained but opposite effects to passing growth cones. Neuron. 1995;14:275–85. 10.1016/0896-6273(95)90285-6 7531986

[43] Courtine G, Gerasimenko Y, van den Brand R, Yew A, Musienko P, Zhong H, et al. Transformation of nonfunctional spinal circuits into functional states after the loss of brain input. Nat Neurosci. 2009;12:1333–42. 10.1038/nn.2401 PMC2828944 19767747

[44] Oatway MA, Chen Y, Bruce JC, Dekaban GA, Weaver LC. Anti-CD11d integrin antibody treatment restores normal serotonergic projections to the dorsal, intermediate, and ventral horns of the injured spinal cord. J Neurosci. 2005;25:637–47. 10.1523/JNEUROSCI.3960-04.2005 PMC6725335 15659600

[45] Falgairolle M, de Seze M, Juvin L, Morin D, Cazalets JR. Coordinated network functioning in the spinal cord: an evolutionary perspective. J Physiol Paris. 2006;100:304–16. 10.1016/j.jphysparis.2007.05.003 17658245

[46] Rowald A, Komi S, Demesmaeker R, Baaklini E, Hernandez-Charpak SD, Paoles E, et al. Activity-dependent spinal cord neuromodulation rapidly restores trunk and leg motor functions after complete paralysis. Nat Med. 2022;28:260–71. 10.1038/s41591-021-01663-5 35132264

[47] Ditunno JF. Outcome measures: evolution in clinical trials of neurological/functional recovery in spinal cord injury. Spinal Cord. 2010;48:674–84. 10.1038/sc.2009.198 20125111

