# Supplementary material for: Intramedullary injection of polymerized laminin in acute traumatic spinal cord injury: a first-in-human pilot study
Source: Spinal Cord. 2026 Aug 4;64(9):834–44. doi: 10.1038/s41393-026-01238-6 (PMC13538055; doi:10.1038/s41393-026-01238-6)
Supplement: Supplementary file 1 [file 41393_2026_1238_MOESM1_ESM.pdf]

Lima et al., 2026

Supplementary files

Figure S1

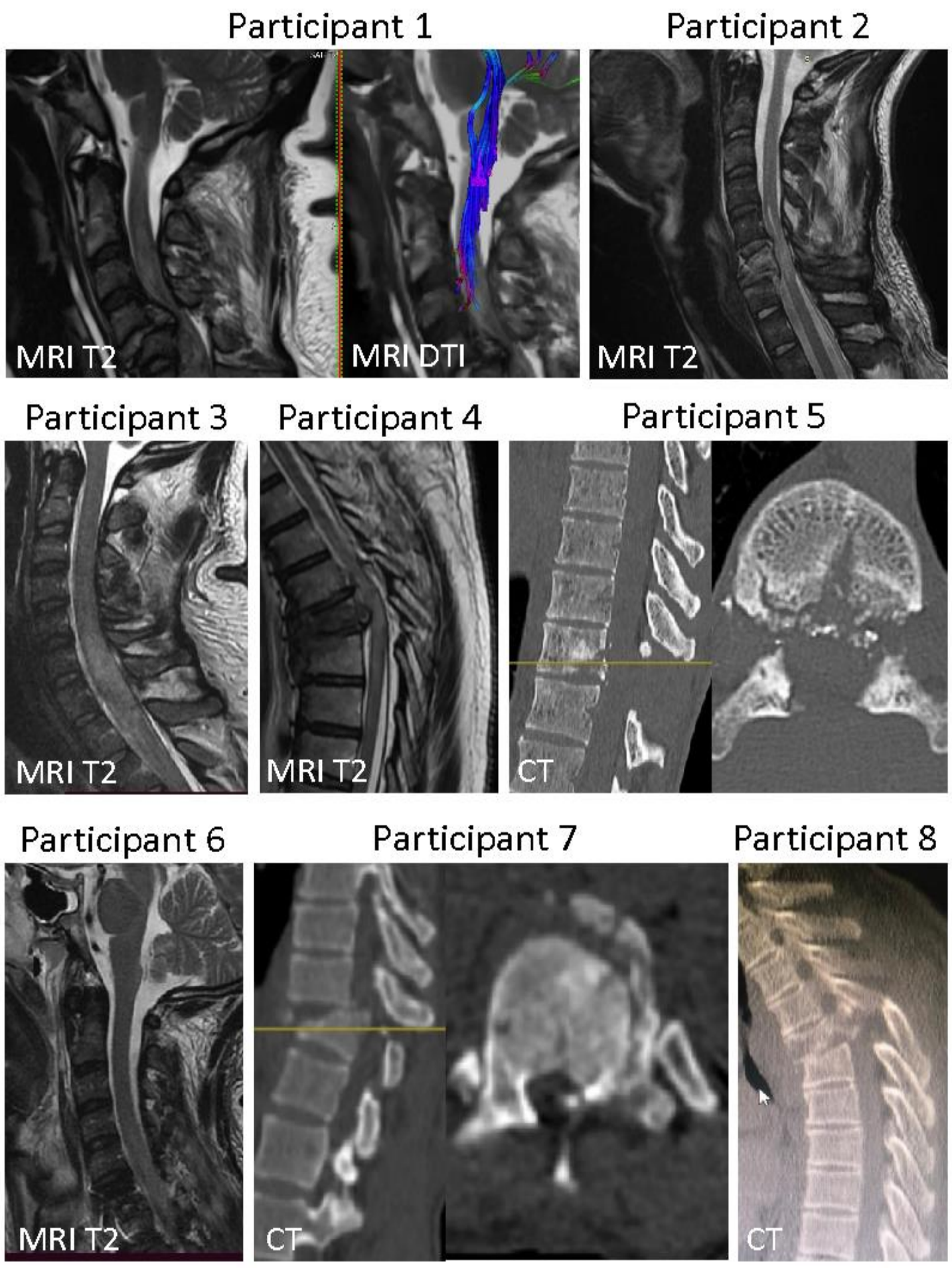

Figure S1. Aspects of the initial lesions observed in MRI or CT. Lesions of participants 1, 2, 3, 4 and 6 were evaluated by MRI. Lesions of participants 5, 7 and 8 were evaluated by CT due to the presence of projectile fragments (participant 5) or because the hospitalization centres had no MRI equipment available (participants 7 and 8) and it would not be advisable to move patients during the COVID-19 pandemic.

Table S1

Study sites	Included participants
**Public Hospitals**	
Public State Hospital Azevedo Lima (HEAL)	1, 8
Army Central Hospital (HCE)	3
Rio de Janeiro Municipal Hospital Souza Aguiar (HMSA)	4, 5[a], 7
**Private Hospitals**	
Hospital Copa Star (HCS)	2
Hospital Ênio Serra (HES)	5[b]
Hospital São José (HSJ)	6[a]
Hospital Vila da Serra (HVS)	6[b]

Table S1. Study sites. [a] hospital of inclusion, [b] hospital of long-term hospitalization

Table S2

**Inclusion criteria**
• Age between 18 and 70 years old<br>• ASIA Impairment Scale grade A<br>• Traumatic spinal cord injury at neurologic level C4 to T12<br>• Written informed consent by patient or legally authorized representative to participate in the study
**Exclusion criteria**
• Severe brain trauma<br>• Need of permanent mechanical respiratory support<br>• Concomitant trauma considered by the study physicians to be severe enough to preclude neurological improvement<br>• Previous neurological diseases (EX. Alzheimer's, Amyotrophic Lateral Sclerosis, Cerebrovascular Accident sequelae) or previous neurological deficits of any aetiology<br>• Abuse or dependence of alcohol or illicit drugs<br>• Any other comorbidity that, at the discretion of the investigator, precludes inclusion of the patient in the study

Table S2. Eligibility criteria.

Table S3

Complication type grouped by organ/system (N= 8)		No. of complications	No. of patients	Incidence
Infection	Pneumonia	3	3	
	Blood stream infection	3	3	
	Intestinal translocation infection	1	1	
	Total of infections	*7*	*5*	63%
Cardiovascular	Arrhythmia<br>Bradycardia	1<br>2	1<br>1	
	Hypovolemic shock	1	1	
	Hypotension	1	1	
	Cardiorespiratory arrest	2	2	
	Cardiac tamponade	1	1	
	Total of cardiovascular complications	*8*	*2*	25%
Pulmonary	Respiratory failure<br>Lobar collapse/atelectasis	2<br>1	1<br>1	
	Haemothorax	2	2	
	Hypoxemia	1	1	
	Total of pulmonary complications	*6*	*3*	38%

Gastrointestinal Genitourinary (GI/GU)	Rhabdomyolysis	3	3	
	Intestinal pseudo-obstruction	1	1	
	Total of GI/GU complications	*4*	*3*	38%
Neurological/ Psychiatric (N/P)	Delirium and agitation	2	2	
	Reduced level of consciousness	1	1	
	Total of N/P complications	*3*	*2*	25%
Haematological	Anaemia	2	2	
	Total of haematological complications	*2*	*2*	25%
Skin	Pressure sores	1	1	
	Total of skin complications	*1*	*1*	17%

Table S3. Severe complications in 8 patients (30 days after SCI).

Table S4

Medical complications		Classification of complication	N. of complications
**Infection**	Urinary tract infection	M (10[2,4,5,6,7,8])	10
	Pyelonephritis	S (1[4])	1
	Pneumonia	S (3[1,6,8]), M (1[3])	4
	Genital Herpes infection	Mi (1[2])	1
	Blood stream infection	S (3[2,5,8]), M(1[3])	4
	Fungal skin infection	Mi (1[2])	1
	Intestinal translocation infection	S (1[8])	1
	Sepsis	S (1 [8])	1
	**TOTAL**		**23**
**Pulmonary**	Respiratory failure	S (2[1])	2
	Lobar collapse/atelectasis	S (1[1]), M (1[4])	2
	Haemothorax	S (2[4, 5])	2
	Hypoxemia	S (1[1])	1
	Pleural effusion	M (1[5])	1
	Desaturation	M (1[5])	1
	**TOTAL**		**9**
**Neurological/ psychiatric**	Pain	Mi (2[1,5]), M(3[2,3,7]),	5
	Delirium and agitation	S (2[1,6]), M (2[2,8])	4
	Headache	Mi (1[2])	1
	Reduced level of consciousness	S (1[1])	1
	Mental confusion	M (1[4])	1
	Dysautonomia	M (1[2])	1
	Vertigo	Mi (2[2,7])	2
	Muscle spasm	M (1[2]), Mi (1[7])	2
	Diabetes insipidus (polyuria)	M (1[6])	1
	Drooling	Mi (1[6])	1
	**TOTAL**		**19**
**Haematological**	Anaemia	S (2[6,8])	2
	**TOTAL**		**2**
**Gastrointestinal and Genitourinary**	Nausea/vomiting	M (1[6])	1
	Gastroparesis	M (1[6])	1
	Rhabdomyolysis	S (3[2,6,8])	3
	Constipation	Mi (5[2,4,5,7])	5
	Intestinal pseudo-obstruction	S(1[8])	1

	Heartburn	Mi (1^{2})	1
	Urinary retention	Mi (1^{2}), M(1^{4})	2
	Pre-renal failure	M (1^{3})	1
	Anuria	M (1^{3})	1
	Haematuria	M (1^{5})	1
	Diarrhoea	Mi (1^{6})	1
	**TOTAL**		**18**
**Skin**	Pressure-damage skin	M ($3^{2,7,8}$), S (1^{4})	4
	Pruritus (pharmacology allergy)	Mi (1^{4})	1
	Surgical wound hyperaemia	Mi (1^{5})	1
	**TOTAL**		**6**
**Cardiovascular**	Arrythmia	S (1^{1})	1
	Bradycardia	S (2^{1})	2
	hypovolemic shock	S (1^{1})	1
	Septic shock	S (1^{8})	1
	Hypotension	Mi(1^{1}), S (1^{1})	2
	Hypertension	M (1^{8})	1
	Cardiorespiratory arrest	S ($3^{1,3,8}$)	3
	Cardiac tamponade	S (1^{3})	1
	**TOTAL**		**12**
**OTHERS**	Arthrodesis equipment poorly adapted by cervical tomography	Mi (1^{1})	1
	Lower limb oedema associated with heterotopic ossification	M (1^{5})	1
	lower limb oedema	M (1^{8})	1
	**TOTAL**		**3**

Table S4. All medical complications. Medical complications observed in the 8 research participants over 12 months of clinical follow-up. The first column describes the types of complications, grouped by organ/system. The second column describes the classification of the complication as mild (Mi), moderate (M) or severe (S). The numbers in parentheses mean the number of events within each classification, and the superscripts identify the participants displaying the event. The third column describes the total number of complications in each category.

Table S5

	Creatinine	Urea	ALT	AST
**Before**				
Normal[1]	7 (88)	8 (100)	3 (75)	1 (20)
Mild[2]	1 (12)	0 (0)	0 (0)	3 (60)
Moderate[3]	0 (0)	0 (0)	1 (25)	1 (20)
Severe[4]	0 (0)	0 (0)	0 (0)	0 (0)
Total	8 (100)	8 (100)	4 (100)	5 (100)
**Two days after**				
Normal	7 (88)	7 (88)	4 (80)	1 (20)
Mild	1 (12)	1 (12)	1 (20)	3 (60)
Moderate	0 (0)	0 (0)	0 (0)	1 (20)
Severe	0 (0)	0 (0)	0 (0)	0 (0)
Total	8 (100)	8 (100)	5 (100)	5 (100)
**Ten days after**				
Normal	7 (88)	8 (100)	2 (60)	1 (20)
Mild	1 (12)	0 (0)	3 (40)	4 (80)
Moderate	0 (0)	0 (0)	0 (0)	0 (0)
Severe	0 (0)	0 (0)	0 (0)	0 (0)
Total	8 (100)	8 (100)	5 (100)	5 (100)

Table S5. Renal and hepatic markers before and after polylaminin administration.
[1]Normal applies to the following reference values: creatinine, 0.5-1.2 mg/dL; urea, 18-55 mg/dL; alanine aminotransferase (ALT), lower than 41 U/dL, and aspartate aminotransferase (AST), lower than 35 U/mL; [2]Mild: > upper limit of normal (ULN) to 2.5 x ULN; [3]Moderate: > 2.5-5 x ULN; [4]Severe: > 5-20 x ULN.

Table S6

Participant		Initial (before treatment)	1 day	2 days	1 month	3 months	6 months	12 months	Reference values
1	Red cells (x10^6/mm^3)	4.29	3.5	3.86					3.9 - 5.1
	Haemoglobin (g/dL)	11.1	9.4	10.8					12.0 - 16
	Haematocrit (%)	34.6	28.2	30.8					35 - 45 %
	Leukocytes (/mm^3)	11,100	12,620	15,690					4,000 – 10,000
	Basophils (%)	0.1	0	0					0 - 2 %
	Eosinophils (%)	0.7	0	0					1 - 6 %
	Neutrophils (%)	84.8	80.0	55					40 - 80 %
	Rods (%)	-	5	1					0 - 5 %
	Segmented (%)	-	75	54					40 - 80 %
	Lymphocytes (%)	11.2	17	40					20 - 50 %
	Monocytes (%)	3.2	3	5					2 - 10 %
	Platelets (/mm^3)	315,000	274,000	283,000					150,000 – 450,000
2	Red cells (x10^6/mm^3)	4.8	3.53	3.27	4.33	4.98	5.13	5.09	4.3 - 5.5
	Haemoglobin (g/dL)	15.1	11.1	10.6	13.9	14.8	16.2	16.1	13.5 - 18.0
	Haematocrit (%)	47.3	34.8	32.5	41.9	43	44.8	46.5	40 - 50%
	Leukocytes (/mm^3)	11,200	12,100	7,600	7,400	5,060	8,480	4,540	4,500 – 13,000
	Basophils (%)	0	0	0	0	1	0.2	0.4	0 - 2 %
	Eosinophils (%)	1	0	2	2	4	0.4	2	1 - 6 %
	Neutrophils (%)	90	83	69	75	55	79.9	49.3	40 - 80 %
	Rods (%)	2	2	2	1	0	-	0	0 - 5 %
	Segmented (%)	88	81	67	74	55	-	49.3	40 - 80 %
	Lymphocytes (%)	6	12	24	15	34	9.4	41.9	20 - 50 %
	Monocytes (%)	3	5	5	8	6	10.1	6.4	2 - 10 %
	Platelets (/mm^3)	167,000	133,000	129,000	126,000	128,000	145,000	118,000	150,000 – 450,000
3	Red cells (x10^6/mm^3)	3.89	3.47	3.55	-	-	-	-	4.3 - 5.5
	Haemoglobin (g/dL)	12.5	11	11.3	-	-	-	-	13.5 - 18.0
	Haematocrit (%)	36.3	31.8	32.3	-	-	-	-	40 - 50%
	Leukocytes (/mm^3)	8,300	6,500	8,000	-	-	-	-	4,500 – 13,000
	Basophils (%)	0	0	0	-	-	-	-	0 - 2 %
	Eosinophils (%)	0	0	2	-	-	-	-	1 - 6 %

	Neutrophils (%)	86	68	65	-	-	-	-	40 - 80 %
	Rods (%)	2	1	1	-	-	-	-	0 - 5 %
	Segmented (%)	86	67	64	-	-	-	-	40 - 80 %
	Lymphocytes (%)	8	27	29	-	-	-	-	20 - 50 %
	Monocytes (%)	4	5	4	-	-	-	-	2 - 10 %
	Platelets (/mm³)	191,000	131,000	146,000	-	-	-	-	150,000 – 450,000
4	Red cells (x10⁶/mm³)	3.27	3.1	2.93	3.15	4.22	4.73	5.04	3.9 - 5.1
	Haemoglobin (g/dL)	9.6	8.7	8.5	8.2	10.5	12.4	13.5	12.0 - 16
	Haematocrit (%)	26.5	25.9	24.4	25.2	32	38.4	43.7	35 - 45 %
	Leukocytes (/mm³)	7,200	8,400	7,600	11,900	4,800	6,900	5,120	4,000 – 10,000
	Basophils (%)	0.1	0	0	0	0	0	1	0 - 2 %
	Eosinophils (%)	1	0	2	5	5	2	2	1 - 6 %
	Neutrophils (%)	78.2	84	74	76	46	60	55	40 - 80 %
	Rods (%)	2	6	6	2	3	3	2	0 - 5 %
	Segmented (%)	75	78	68	74	43	57	53	40 - 80 %
	Lymphocytes (%)	15	10	20	13	36	27	36	20 - 50 %
	Monocytes (%)	7	6	4	6	13	11	6	2 - 10 %
	Platelets (/mm³)	123,000	83,000	124,000	312,000	229,000	210,000	222,000	150.000 - 450.000
5	Red cells (x10⁶/mm³)	3.3	2.63	2.36	4.04	-	4.8	4.56	4.3 - 5.5
	Haemoglobin (g/dL)	9.7	7.7	7.5	11.6	-	13.4	13.2	13.5 - 18.0
	Haematocrit (%)	29.2	23.6	20.9	35	-	42.7	39	40 - 50%
	Leukocytes (/mm³)	10,800	8,400	7,200	6,800	-	3,700	10,800	4,500 – 13,000
	Basophils (%)	0	0	0	0	-	0	0	0 - 2 %
	Eosinophils (%)	0	2	1	2	-	2	3	1 - 6 %
	Neutrophils (%)	90	73	75	64	-	46	65	40 - 80 %
	Rods (%)	1	3	2	3	-	-	2	0 - 5 %
	Segmented (%)	89	70	73	61	-	-	63	40 - 80 %
	Lymphocytes (%)	3.1	20	18	29	-	47	25	20 - 50 %
	Monocytes (%)	6.9	5	6	5	-	5	7	2 - 10 %
	Platelets (/mm³)	162,000	115,000	123,000	447,000	-	307,000	315,000	150,000 – 450,000
6	Red cells (x10⁶/mm³)	2.73	2.91	3.4	3.73	3.66	4.22	4.59	4.3 - 5.5
	Haemoglobin (g/dL)	7.6	8.2	9.4	10.4	10.2	12.19	13.1	13.5 - 18.0
	Haematocrit (%)	24	25.9	29.5	33.1	31.6	39.5	40.4	40 - 50%

	Leukocytes (/mm³)	5,000	7,020	5,610	7,020	4,630	4,770	4,720	4,500 – 13,000
	Basophils (%)	0	0	0	0	0.5	0.2	0.2	0 - 2 %
	Eosinophils (%)	0	1	4.6	2	8.4	3.8	4.7	1 - 6 %
	Neutrophils (%)	89	-	-	-	43.5	-	-	40 - 80 %
	Rods (%)	5	-	-	-	-	-	-	0 - 5 %
	Segmented (%)	84	83.2	66.7	60	-	54.9	53.4	40 - 80 %
	Lymphocytes (%)	9	11.1	20.7	26	38.9	32.7	31.8	20 - 50 %
	Monocytes (%)	2	4.7	8	12	8.7	8.4	10	2 - 10 %
	Platelets (/mm³)	123,000	128,000	155,000	248,000	218,000	206,000	193,000	150,000 – 450,000
7	Red cells (x10⁶/mm³)	3.6	2.9	2.9	3.21	4.7	4.14	4.34	3.9 - 5.1
	Haemoglobin (g/dL)	11	8.8	8.9	11.3	12.8	12	13.4	12.0 - 16
	Haematocrit (%)	33.8	28.7	28.8	32.7	40.6	35.7	39.6	35 - 45 %
	Leukocytes (/mm³)	8,500	10,500	9,300	10,750	10,000	13,180	9,370	4,000 – 10,000
	Basophils (%)	0	0	0	0	0	0	0.2	0 - 2 %
	Eosinophils (%)	0	0	0	3	8	1	1.3	1 - 6 %
	Neutrophils (%)	82	70	97	65	57	85	64.4	40 - 80 %
	Rods (%)	3	3	27	2	0	3	-	0 - 5 %
	Segmented (%)	79	67	70	65	57	82	-	40 - 80 %
	Lymphocytes (%)	12	18	2	23	28	12	26.7	20 - 50 %
	Monocytes (%)	6	12	10.1	7	7	2	7.4	2 - 10 %
	Platelets (/mm³)	244,000	266,000	227,000	349,000	330,000	316,000	259,000	150,000 – 450,000
8	Red cells (x10⁶/mm³)	4.31	-	3.37	3.19				4.3 - 5.5
	Haemoglobin (g/dL)	13.3	-	10.6	9.7				13.5 - 18.0
	Haematocrit (%)	38.8	-	30.4	26.3				40 - 50%
	Leukocytes (/mm³)	11,370	-	13,160	26,030				4,500 – 13,000
	Basophils (%)	2	-	0	0				0 - 2 %
	Eosinophils (%)	0	-	2	0				1 - 6 %
	Neutrophils (%)	110	-	83	89				40 - 80 %
	Rods (%)	3	-	3	7				0 - 5 %
	Segmented (%)	80	-	80	82				40 - 80 %
	Lymphocytes (%)	11	-	10	8				20 - 50 %
	Monocytes (%)	4	-	5	3				2 - 10 %
	Platelets (/mm³)	133,000	-	101,000	435,000				150,000 – 450,000

Table S6. Blood counts before and after polylaminin administration.
